# Cytochrome P450 gene family: cross-pathway functional conservation, novel catalytic reactions, and synthetic biology-driven applications in plant secondary metabolism

**DOI:** 10.3389/fpls.2026.1765290

**Published:** 2026-02-20

**Authors:** Lang Chen, Yingying Zhao, Sujing He, Jialing Lei, Hongwei Li, Zhizhai Liu, Liang Zhang, Liwen Yang, Kuanping Deng, Runlan Wan, Delin Xu

**Affiliations:** 1 Department of Medical Instrumental Analysis, School of Preclinical Medicine of Zunyi Medical University, Zunyi, Guizhou, China; 2 Basic Medical Sciences Center for Integrated Research and Practical Innovation, School of Preclinical Medicine of Zunyi Medical University, Zunyi, Guizhou, China; 3 Zunyi Academy of Agricultural Sciences, Zunyi, Guizhou, China; 4 College of Agronomy and Biotechnology of Southwest University, Chongqing, China; 5 School of Food and Bioengineering, Food Microbiology Key Laboratory of Sichuan Province, Xihua University, Chengdu, China; 6 School of Medicine, Southwest Jiaotong University, Chengdu, China; 7 The Affiliated Hospital of Southwest Medical University, Luzhou, Sichuan, China

**Keywords:** biosynthesis, CYPs, cytochrome P450 enzymes, engineering application, gene regulation, secondary metabolites

## Abstract

Plant secondary metabolites play fundamental roles in plant defense and environmental adaptation, and possess extensive high-value applications in medicine, agriculture, and industrial biotechnology. The cytochrome P450 (CYPs) family occupies a central position in metabolic networks by catalyzing key reactions in the biosynthesis of terpenoids, alkaloids, and flavonoids. Although the role of CYPs in these pathways is well documented, their precise catalytic mechanisms and regulatory networks remain poorly characterized. In this review, we summarize recent advances in CYP classification, structural features, and catalytic diversity across plant species. We also analyze the transcriptional regulation and environmental signals that control *CYP* gene expression. Based on this synthesis, we propose an integrated strategy combining CYP enzyme engineering with metabolic pathway optimization to enhance the sustainable production of valuable secondary metabolites. Furthermore, we outline how CYP-centered approaches can improve the quality of medicinal plants and enable scalable bioreactor-based production. Interdisciplinary collaboration, supported by emerging technologies such as synthetic biology and machine learning, will be essential to overcome current limitations in CYP functional characterization, providing both mechanistic insights and practical solutions for the large-scale production of plant-derived natural products.

## 1 Introduction

Secondary metabolites (SMs) are a chemically diverse group of small molecules produced through complex metabolic pathways. These compounds play essential roles in plant defense and environmental adaptation. Due to their broad applications in pharmaceuticals, cosmetics, flavorings, and agricultural products, SMs have become a major focus of modern research. Understanding and harnessing the catalytic mechanisms of the enzymes involved in SM biosynthesis offer the most direct and sustainable approach to enhancing their production. However, with growing global demand, the gap between high market needs and the naturally low accumulation of SMs in plants has intensified the urgency of leveraging key biosynthetic enzymes for scalable and efficient biomanufacturing (Abdulhafiz et al., 2022). Cytochrome P450 enzymes (CYPs) are primary mono-oxygenases central to plant specialized metabolism. They catalyze the regio- and stereoselective incorporation of oxygen into aliphatic or aromatic carbon structures, producing key intermediates such as ketones and alcohols. These intermediates are essential for forming the core scaffolds of terpenoids, alkaloids, and flavonoids (Ackah et al., 2024; Forman et al., 2024; Guo et al., 2025). Plant secondary metabolites and plant hormones are also functionally interconnected through CYP enzymes. CYPs link primary and secondary metabolism. For example, Baeyer-Villiger oxidation mediated by CYP85A2 participates in the biosynthesis of brassinosteroids, while CYP enzymes from the same family also catalyze modifications of terpenes and flavonoids for defense. This dual function enables plants to coordinate growth and defense, while CYP-derived secondary metabolites enhance stress resistance, forming an integrated metabolic network (Nomura et al., 2005). Therefore, a comprehensive understanding of CYP catalytic mechanisms, combined with strategic enzyme engineering, is critical for the efficient and sustainable production of these valuable metabolites.

**Figure 1:**
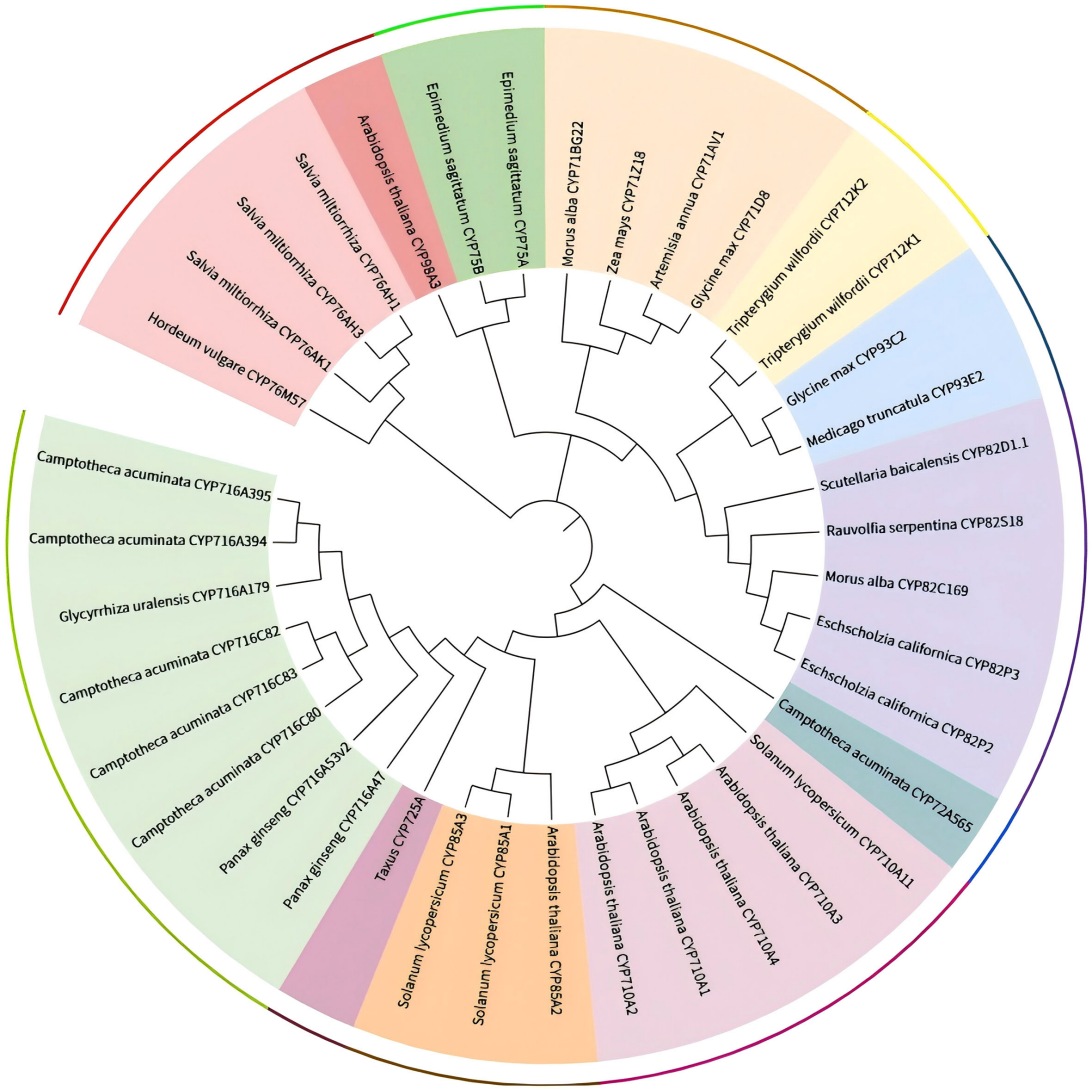
Phylogenetic tree of the cytochrome P450 family involved in the synthesis of some secondary metabolites.

The cytochrome P450 family is central to the structural diversification of plant secondary metabolites, a role supported by its extensive genetic repertoire, structural conservation, catalytic versatility, and broad substrate acceptance. Since the first identification of plant *CYP* genes in 1962, more than 300,000 CYP sequences have been annotated in public databases, distributed across over 670 families (Hansen et al., 2021). Functioning as heme-thiolate monooxygenases (Baader and Meyer, 2007; Mokhosoev et al., 2024), CYPs form the largest enzyme family in plants (Xu et al., 2015),and participate in both the synthesis and turnover of SMs, greatly expanding the structural diversity of metabolic outcomes (Mai et al., 2024). This functional versatility establishes CYPs as key regulatory nodes within plant specialized metabolism. We summarized the CYP450 family involved in the synthesis of terpenoids, flavonoids, alkaloids, and other types of secondary metabolites. With reference to existing literature, we compiled some important CYP450 family genes involved in these secondary metabolic pathways. According to the CYP450 subfamily classification, we constructed a phylogenetic tree using MEGA software and beautified it through the iTOL website. The results are shown in **Figure 1**. However, substantial knowledge gaps remain regarding their precise catalytic mechanisms, regulatory networks, and functional divergence among species. Critical challenges—such as narrow substrate specificity, inefficient electron transfer, and heterologous expression bottlenecks—continue to limit the scalable production of valuable SMs.

In light of the pivotal role of CYPs in SM biosynthesis, a systematic review of their molecular mechanisms and recent advances is necessary. Examining current engineering challenges and future opportunities for CYP applications can inform more efficient bio-based production strategies. This review synthesizes recent literature to explore CYP-involved regulatory networks and pathway interactions, highlighting persistent obstacles in enzymatic functionality, electron transport, and heterologous expression. We further discuss emerging synthetic biology approaches for enhancing CYP activity, optimizing bioreactor design, and building CYP-centered metabolic engineering platforms. By elucidating CYP regulatory mechanisms and proposing novel engineering strategies, this work aims to support the sustainable industrial production of plant secondary metabolites.

## 2 Elucidating the modulatory networks of plant CYPs in secondary metabolism

**Figure 2:**
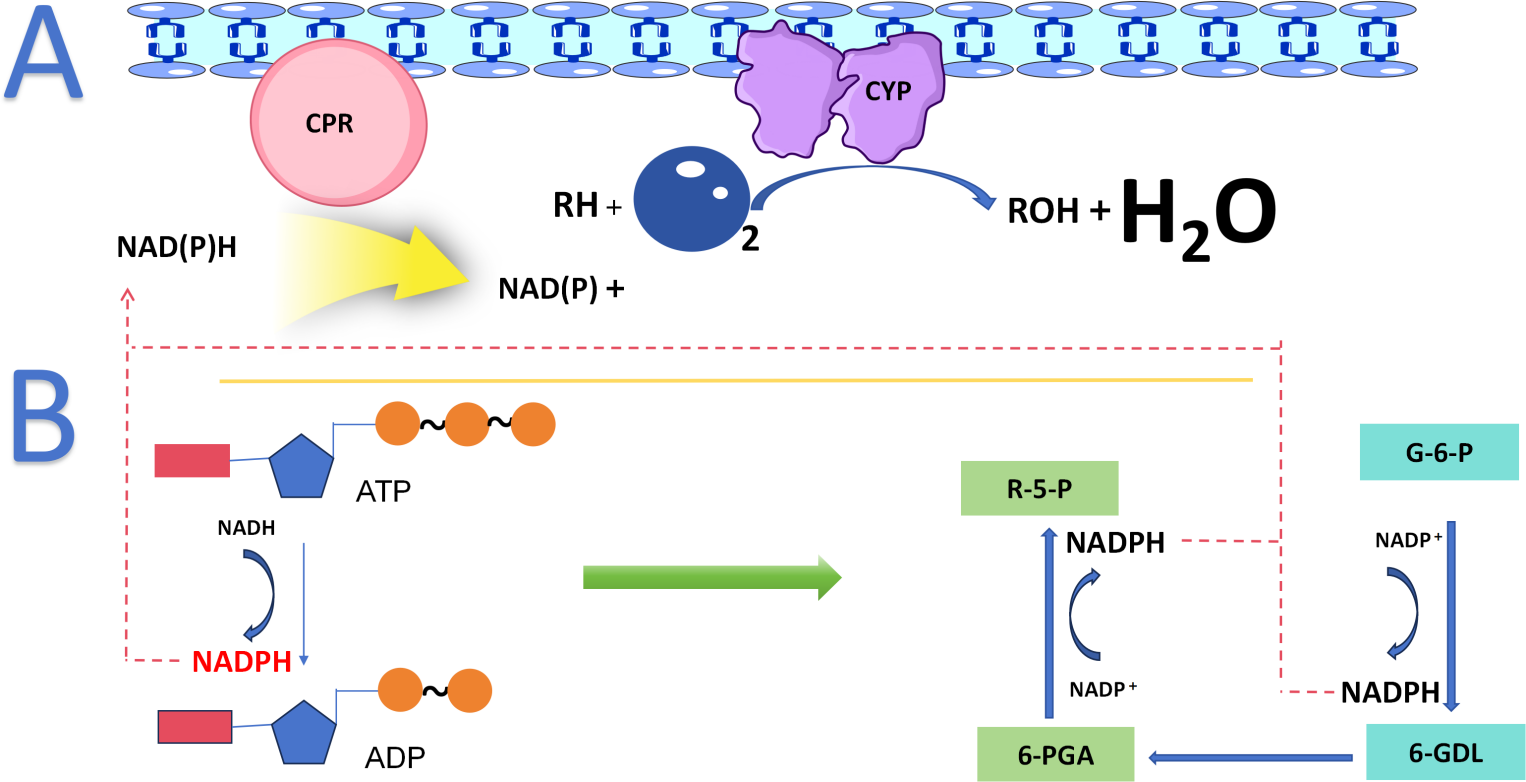
CYP Enzyme System and Catalytic Core in Plant Secondary Metabolism. **(A)** CYP enzymes interact with CPR, using NAD(P)H to drive substrate oxidation. **(B)** The pentose phosphate pathway and ATP hydrolysis provide energy for CYP reactions.

The majority of plants CYPs reside in the endoplasmic reticulum (ER). Their structure features several highly conserved catalytic motifs, including the heme-binding domain FxxGxRxCxG, the K-helix motif ExxR, the PERF motif PxRx, and the I-helix motif AGxD/ET. These conserved motifs confer on CYPs a broad and robust catalytic versatility, enabling them to perform regio- and stereoselective oxidations of C-C and C-H bonds under mild conditions. As monooxygenases, CYPs catalyze the incorporation of an oxygen atom from O_2_ into a substrate, a process that depends on a dedicated electron transfer chain (Zou et al., 2021) (**Figure 2**). The remarkable diversity of the CYP gene family, along with its broad catalytic repertoire, drives the production of a wide array of metabolic compounds. This establishes CYPs as central hubs in the regulatory networks of plant secondary metabolism (Singh et al., 2021; Malhotra and Franke, 2022; Mi et al., 2023; Nie et al., 2024).

### 2.1 Biosynthetic pathways of plant secondary metabolites

Terpenoids are classified based on the number of isoprene units into major subclasses, which include monoterpenes, sesquiterpenes, diterpenes, triterpenes, and polyterpenes. Their biosynthesis is primarily governed by two fundamental metabolic routes: the mevalonate (MVA) pathway and the methylerythritol phosphate (MEP) pathway. The MVA pathway occurs in the cytoplasm, where acetyl-CoA serves as the initial substrate for producing the essential C5 precursors—isopentenyl pyrophosphate (IPP) and dimethylallyl pyrophosphate (DMAPP). In contrast, the MEP pathway takes place in plastids, utilizing glyceraldehyde-3-phosphate and pyruvate to generate IPP and DMAPP. This compartmentalization is critical for regulating the temporal and spatial production of diverse terpenoid compounds (Nabavi et al., 2020; Kant et al., 2024).

Flavonoids possess a characteristic C6–C3–C6 skeleton, consisting of two aromatic benzene rings (A and B) linked by a three-carbon bridge. In plants, their biosynthesis derives from carbon units supplied by both the acetyl-CoA and shikimate–phenylpropanoid pathways. Chalcone synthase (CHS) catalyzes the condensation of malonyl-CoA with p-coumaroyl-CoA to form the core chalcone structure. This intermediate is subsequently isomerized by chalcone isomerase (CHI) into flavanone, which can be further modified into various flavonoid subclasses, such as isoflavones and anthocyanins, by multiple downstream enzymes (Liu et al., 2021).

Alkaloid biosynthesis typically begins with specific amino acid derivatives. For example, tyramine, a derivative of tyrosine metabolism, reacts with 3,4-dihydroxybenzaldehyde to form norbelladine. This key intermediate then undergoes sequential methylation and oxidative modifications to yield bioactive alkaloids such as galanthamine and colchicine (Tariq et al., 2023).

The biosynthetic pathways of terpenoids, flavonoids, and alkaloids are core components of the plant secondary metabolic network. CYPs pivotally catalyze key oxidative and modification reactions in these pathways, significantly enhancing the structural diversity of plant metabolites. Understanding these enzymatic functions enhances our knowledge of plant metabolism and provides a theoretical foundation and technical platform for developing bioactive compounds from medicinal plants and advancing metabolic engineering strategies. Further investigation into the precise catalytic and regulatory mechanisms of CYPs will facilitate the sustainable and scalable production of valuable plant-derived natural products.

### 2.2 CYPs regulate terpenoid biosynthesis

Terpenoids are one of the most structurally diverse and chemically complex classes of primary and secondary metabolites in nature, playing crucial roles in plant biology (Bergman et al., 2024). This structural diversity underlies their significant research value and broad potential for applications, particularly in pharmacology and biotechnology, where they exhibit a range of bioactive properties such as antioxidant, anticancer, and immunomodulatory effects. Additionally, terpenoids are essential for plant growth, defense, and environmental adaptation (Nagegowda and Gupta, 2020; Zhang et al., 2023; Zhang et al., 2025).

**Figure 3:**
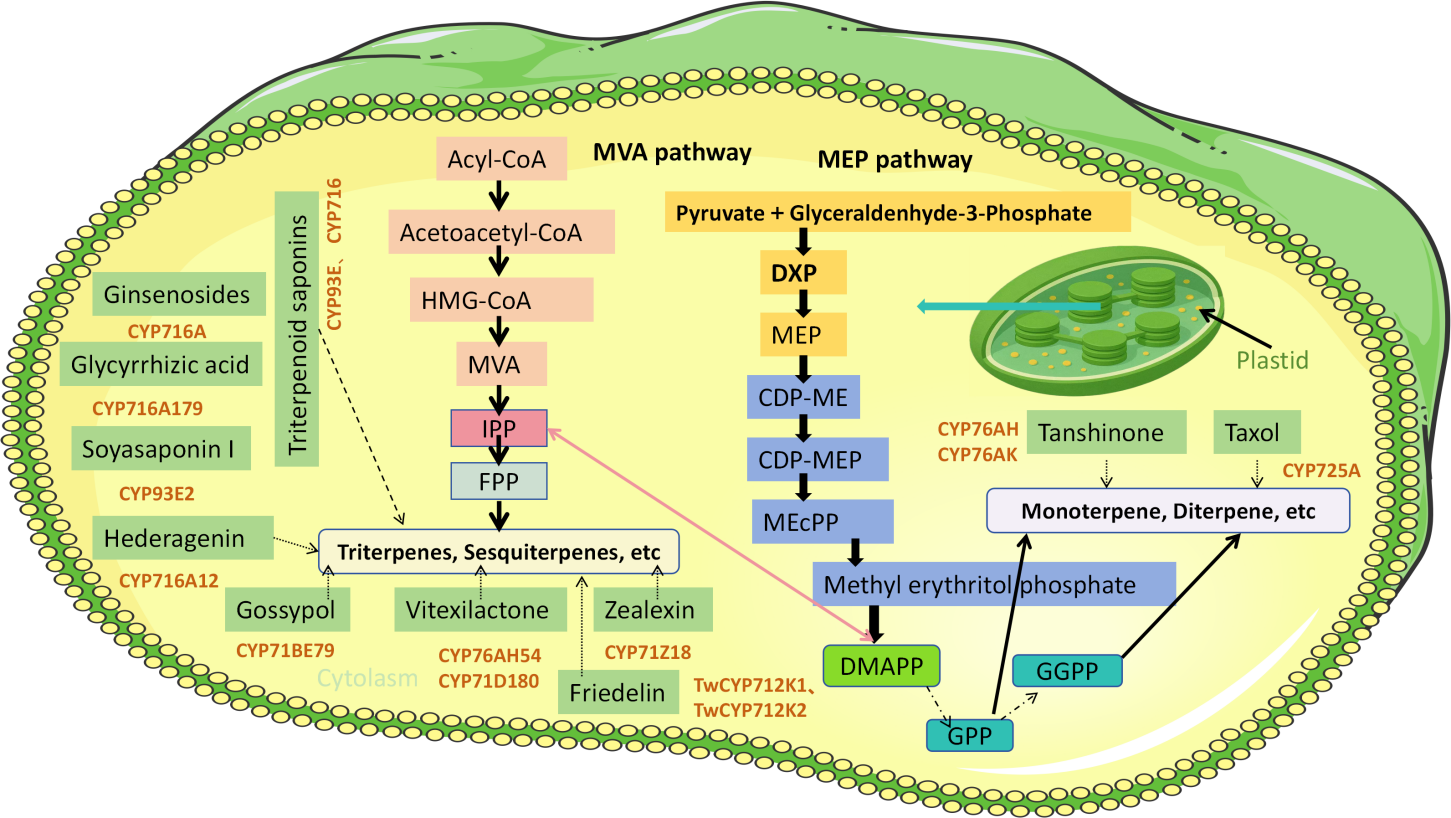
CYPs Regulate the metabolic network of terpenoid compound biosynthesis in plants. MVA, Mevalonate; MEP, Methylerythritol Phosphate; IPP, Isopentenyl Pyrophosphate; DMAPP, Dimethylallyl Pyrophosphate; DXP, 1-Deoxy-D-xylulose 5-phosphate; CDP-ME, 4-(Cytidine 5′-diphospho)-2-C-methyl-D-erythritol; CDP-MEP, 4-(Cytidine 5′-diphospho)-2-C-methyl-D-erythritol 2-phosphate; MEcPP, 2-C-Methyl-D-erythritol 2,4-cyclodiphosphate; FPP, Farnesyl Pyrophosphate; GPP, Geranyl Pyrophosphate; GGPP, Geranylgeranyl Pyrophosphate.

To date, multiple cytochrome P450 subfamilies have been identified as key catalysts in the biosynthesis of plant terpenoids (**Figure 3**). These CYP enzymes mediate critical oxidative, hydroxylative, and cyclization reactions that modify the terpenoid scaffold, thereby greatly expanding structural diversity. For example, members of the CYP76 and CYP71 subfamilies contribute to the biosynthesis of diterpenoids such as gibberellins and paclitaxel precursors. The functional specificity of these enzymes is largely determined by the spatial configuration and chemical microenvironments of their active sites, which dictate substrate recognition and catalytic efficiency (Bathe and Tissier, 2019).

#### 2.2.1 Oxidation-driven scaffold functionalization in terpenoids

Plants have evolved a highly sophisticated oxidative system centered on cytochrome P450 enzymes (CYPs), which catalyze the site-specific functionalization of terpenoid scaffolds. This activity drives the diversification of defensive and medicinal terpenoids, yielding structures with vast functional differences. Thus, targeting key CYP enzymes presents a promising strategy for enhancing plant resistance or improving the yield of valuable medicinal terpenoids, and provides a foundation for exploring and utilizing the vast repertoire of plant-derived natural products. In *Gossypium hirsutum*, the transcription factor GhMYC2 positively regulates gossypol biosynthesis by directly activating the expression of CYP71BE79. This enzyme is putatively involved in oxidizing gossypol precursors, and its downregulation via gene silencing significantly reduces gossypol accumulation, underscoring its essential role in cotton’s defense metabolism (Han et al., 2022). Similarly, in *Zea mays*, the biosynthesis of zealexins—important antibacterial diterpenoid phytoalexins produced in response to pathogen infection—relies on CYP71Z18. Mao et al. demonstrated that CYP71Z18 catalyzes key oxidative steps in the zealexin pathway, such as the oxidation of dolabradiene, and that loss of its function results in a marked decrease in zealexin levels, thereby compromising maize’s chemical defense response (Mao et al., 2016). In medicinal plants, CYPs also serve as pivotal enzymes in shaping high-value terpenoid scaffolds. For instance, CYP716A179 in *Glycyrrhiza uralensis* specifically catalyzes the C-28 oxidation of the triterpenoid β-amyrin, generating a direct precursor of glycyrrhizic acid (Ghosh, 2017). In *Tripterygium wilfordii*, CYP712K subfamily members TwCYP712K1 and TwCYP712K2 specifically hydroxylate the C-3 position of friedelin to produce a precursor of the anticancer compound triptolide. Moreover, the recently identified CYP712K3 contributes to the accumulation of other defense-related metabolites by oxidizing diterpenoid intermediates (Pei et al., 2021). Most notably, in the artemisinin biosynthetic pathway of *Artemisia annua*, CYP71AV1 plays a central catalytic role by mediating the sequential oxidation of amorpha-4,11-diene to artemisinic acid, a key biosynthetic precursor of artemisinin (Chen et al., 2021).

#### 2.2.2 Hydroxylation as a key driver of terpenoid diversification

Extensive studies have demonstrated that specific cytochrome P450 enzymes precisely catalyze key oxidative modifications of terpenoid scaffolds, directly shaping the structural configuration and biological activity of the resulting metabolites. In *Camptotheca acuminata*, a newly characterized CYP716 subfamily member, CYP716C49, was identified as catalyzing the critical C-16β-hydroxylation step in pentacyclic triterpene biosynthesis. This reaction converts oleanolic acid into a key intermediate for downstream pathways, and overexpression of CYP716 has been shown to significantly enhance the accumulation of the target compound (Pu et al., 2023).

The CYP450 enzyme family plays a pivotal role in mediating terpenoid biosynthesis in medicinal plants, enabling the formation of structurally diverse compounds with significant pharmacological properties. In *Salvia miltiorrhiza Bunge*, CYP76AH1 catalyzes the C-11 and C-12 hydroxylation of miltiradiene, generating precursors essential for the synthesis of salviaquinone derivatives (Mao et al., 2020). Triterpene saponins are a group of biologically active compounds, triterpene saponins are highly structurally diverse, show a wide range of pharmacological activities, and can exert anti-inflammatory effects by inhibiting the NF-κB signaling pathway, and paclitaxel inhibits cancer cell division by stabilizing microtubules, treating Alzheimer’s disease, atherosclerosis, anti-cancer, analgesia, etc (Zafar et al., 2018; Gong et al., 2023; Zhang et al., 2023; Câmara et al., 2024; Lv et al., 2024). Comprehensive characterization of CYP enzymes involved in triterpenoid saponin biosynthesis may facilitate their industrial-scale production and enhance their pharmaceutical utility.CYP93E, CYP716, and related families catalyze oxidation and glycosylation reactions during early triterpenoid saponin biosynthesis. For example, CYP716A12 catalyzes the conversion of oleanolic acid to hederagenin—a reaction transcriptionally regulated by MYB transcription factors (Xu et al., 2021). Similarly, CYP93E2 in *Medicago truncatula* catalyzes the C-24 hydroxylation of β-amyrin to yield sapogenol, a key branching point in saponin biosynthesis. CRISPR-mediated knockout of CYP93E2leads to a significant reduction in saponin content, underscoring its essential role in this pathway (Confalonieri et al., 2021). Furthermore, studies in *Glycyrrhiza uralensis* and *Panax ginseng* have highlighted the central function of the CYP716 family. In ginseng, CYP716A subfamily members catalyze the C-12 and C-6 hydroxylation of dammarenediol-II to produce protopanaxadiol (PPD) and protopanaxatriol (PPT), respectively—core intermediates in ginsenoside biosynthesis (Gwak et al., 2019).

Notably, oxidative and hydroxylative reactions can act synergistically to promote terpenoid biosynthesis. Heskes et al. reported that in *Vitex agnus-castus*, CYP76AH54 and CYP71D180 catalyze multi-step hydroxylation of clerodane-type diterpene precursors at C-6β and C-16, ultimately forming the anti-inflammatory diterpene vitexilactone. This functional synergy depends on their specific expression in glandular trichomes and integration with upstream terpenoid synthases within the metabolic pathway (Heskes et al., 2018). Mao et al. further showed that combining the hydroxylation activity of CYP76AH with the oxidation activity of CYP76AK in *Salvia miltiorrhiza Bunge* enabled the construction of a synthetic biology system that significantly increased salvinone yield, highlighting the importance of multi-enzyme synergy in diterpene biosynthesis (Mao et al., 2020). In the paclitaxel biosynthetic pathway, CYP725A (previously classified as CYP76AH) catalyzes the C-5α hydroxylation of taxadiene to yield taxadien-5α-ol, a key precursor of paclitaxel (Jennewein et al., 2003). CYP725A4 converts the substrate into the novel cyclic ether compound 5(12)-oxa-3(11)-cyclotaxane through a series of oxidation and cyclization steps. This product features a fused five-ring system and an inter-molecular C-5/C-12 ether bridge, providing a new branch in the biosynthesis of taxane compounds (Rontein et al., 2008).

Systematic identification and functional analysis of key CYP450 enzymes involved in medicinal terpenoid biosynthesis—particularly those catalyzing rate-limiting oxidative steps—are critically important. Elucidating their catalytic mechanisms, regulatory networks, and functional synergies will not only provide a solid theoretical foundation but also empower metabolic engineering strategies for the efficient and scalable production of high-value plant-derived pharmaceuticals.

### 2.3 CYPs regulate flavonoid biosynthesis

**Figure 4:**
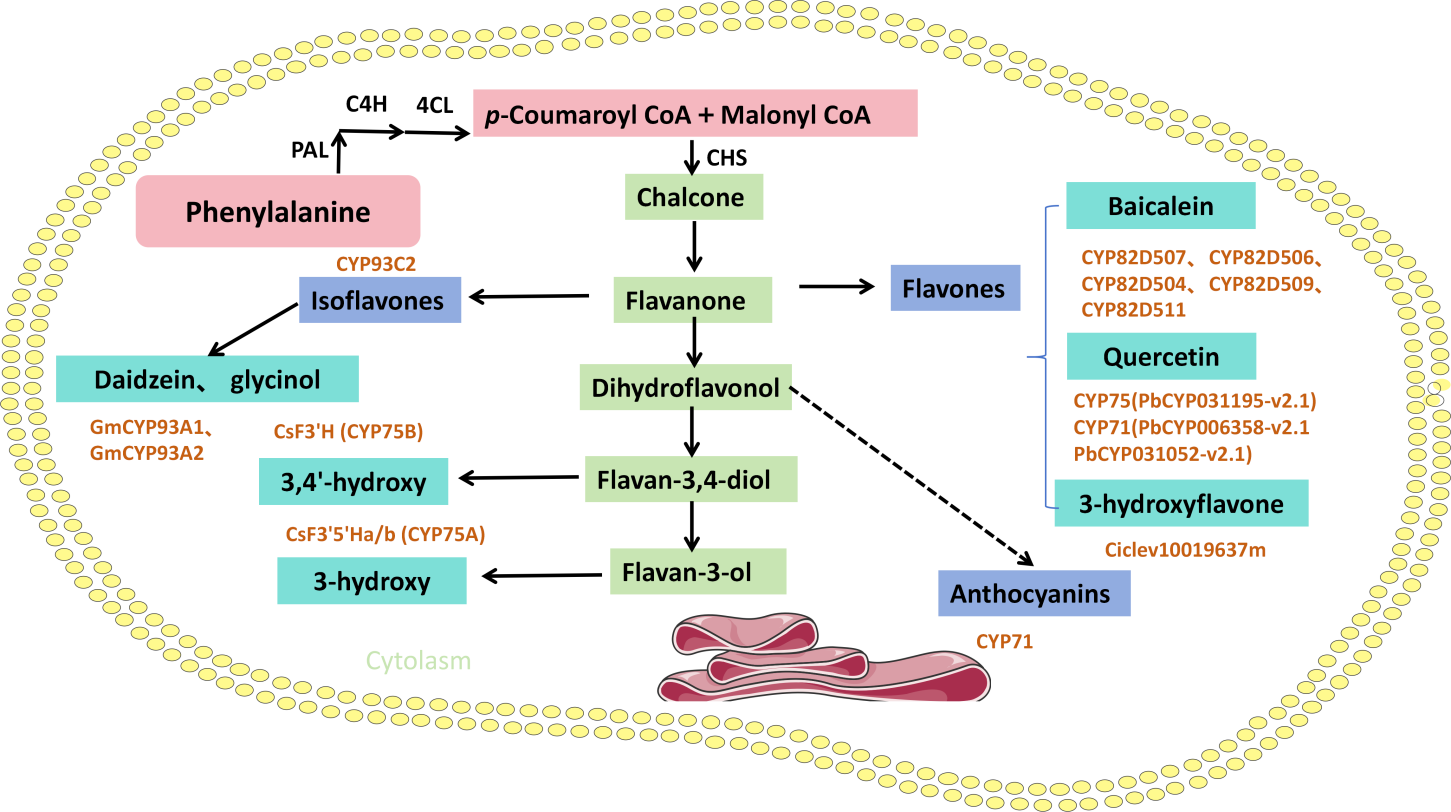
CYP-Mediated synthesis of plant flavonoids.

Flavonoids are natural polyphenolic compounds that serve as the primary pigments in plants. This group includes anthocyanins (imparting red, orange, blue, and purple hues), along with chalcones and aurones (yellow pigments), and flavanols and flavones (white to pale yellow pigments). These compounds exhibit a wide range of pharmacological activities, including antioxidant, anti-inflammatory, antibacterial, and antihypertensive effects. As secondary metabolites, flavonoids possess both nutritional and therapeutic value. The cytochrome P450 family functions as a central molecular determinant of flavonoid chemical diversity across the plant kingdom. This functional diversity is achieved through mechanisms such as tissue-specific expression, developmental regulation, narrow substrate specificity, and site-selective catalysis (**Figure 4**).

#### 2.3.1 CYPs mediate the hydroxylation of flavonol synthesis

Flavonols, a major and widely distributed subgroup of flavonoids, have biological activity and pharmacological potential that are critically determined by their structural modifications. In *Solanum tuberosum*, genome-wide analysis identified 253 *CYP* genes, seven of which were confirmed as key participants in the flavonoid biosynthetic pathway. Within the SiC4H clade (Soltu.DM.06G032850/C4H1 and Soltu.DM.06G032860/C4H2), C4H1 exhibited consistently high transcript levels during red and purple tuber development (stages S1–S3), whereas C4H2 expression peaked in mature tubers and fruits. The SiF3H gene family (F3H1, F3H2, F3H3) displayed distinct spatiotemporal expression patterns: F3H1 was strongly expressed in yellow tubers at stage S2, F3H2 was specifically upregulated in red tubers at stage S3, and F3H3 was preferentially expressed in red tubers. SiF3′5′H (Soltu.DM.11G020990) demonstrated a progressive increase in expression from S1 to S3 in red and purple tuber tissues, thereby directing metabolic flux into the anthocyanin biosynthetic branch. Additionally, SiFNS II (Soltu.DM.05G019180) showed elevated expression in red and purple-skinned tubers at stage S1, contributing to the early regulation of the flavonoid pathway (Li et al., 2024). Collectively, these findings reveal a developmentally regulated, CYP-mediated network governing tuber pigmentation and flavonol composition.

In *Camellia sinensis*, CsF3′H (CYP75B) plays a central role in flavanol metabolism by catalyzing the conversion of 4′-hydroxylated substrates into 3′,4′-dihydroxylated products, whereas CsF3′5′Ha/b (CYP75A) mediates the 3-hydroxylation of catechins. The coordinated action of these enzymes shapes tissue-specific flavonoid accumulation patterns, ultimately determining tea flavor profiles and antioxidant capacity (Guo et al., 2019).

#### 2.3.2 CYPs mediate hydroxylation of flavonoid synthesis

Hydroxylation at specific positions on the flavonoid scaffold is a critical enzymatic step that determines the structural and functional properties of the final compounds. In *Pyrus* spp., Zhang et al. identified PbCYP031195-v2.1, a CYP75 family member encoding flavonoid 3′-hydroxylase (F3′H). This enzyme catalyzes the conversion of dihydrokaempferol and kaempferol to dihydroquercetin and quercetin, respectively, marking the committed step in the biosynthesis of rutin and myricetin (Zhang et al., 2023). Additionally, two CYP71 family members—PbCYP006358-v2.1 and PbCYP031052-v2.1—were functionally annotated as flavonoid 3-hydroxylases (F3H) and participate in the hydroxylation of the flavonoid backbone. In *Scutellaria baicalensis*, the CYP82D subfamily contributes to the structural diversity of bioactive flavones such as baicalin and wogonin, primarily through site-specific hydroxylation reactions, often coupled with methylation. Gene duplication events have led to functional divergence among these enzymes: CYP82D507, CYP82D506, and CYP82D504 predominantly catalyze 8-hydroxylation to yield baicalein, whereas CYP82D509 and CYP82D511 mediate 6-hydroxylation to produce baicalin. Notably, CYP82D511 was recently identified to possess 7-O-demethylase activity, a novel catalytic function. These findings provide a molecular framework for designing heterologous biosynthetic pathways for Scutellaria-derived compounds (Qiu et al., 2025). In *Citrus* spp., Liu et al. first characterized a UV-B-inducible flavonoid 3′-hydroxylase, CitF3′H (Ciclev10019637m). This enzyme catalyzes the 3′-hydroxylation of multiple flavonoid substrates. Gene silencing experiments demonstrated that suppression of CitF3′H significantly reduced the levels of 3′-hydroxylated flavonoids in citrus seedlings, confirming its essential role in flavonoid biosynthesis and composition (Liu et al., 2023). Collectively, these findings demonstrate that plant cytochrome P450 enzymes regulate flavonoid metabolic flux through precise positional hydroxylation and strict substrate selectivity.

The CYP75A/B subfamily plays a particularly important role in determining B-ring hydroxylation patterns, which in turn govern pigment diversity and secondary metabolite profiles. In *Epimedium sagittatum*, EsF3′H (CYP75B) and EsF3′5′H (CYP75A) catalyze 3′-monohydroxylation and 3′,5′-dihydroxylation of the B-ring, respectively, leading to the formation of cyanidin- and delphinidin-type anthocyanins. These reactions are key determinants of anthocyanin composition and thus regulate plant pigmentation (Huang et al., 2012).

#### 2.3.3 CYPs are involved in branching reactions in isoflavone synthesis

Isoflavonoids are a significant subclass of flavonoids predominantly found in legumes (*Leguminosae*), possessing diverse biological activities including antioxidant, anti-inflammatory, and estrogen-like effects. In *Glycine max*, the major isoflavonoids include daidzein, genistein, and glycinol. The isoflavone scaffold is synthesized by the cytochrome P450 enzyme 2-hydroxyisoflavanone synthase (CYP93C). In CYP93C2, the catalytic residues Ser-310 and Lys-375 mediate the aryl migration of flavone substrates (Sawada and Ayabe, 2005), providing the first mechanistic insights into CYP93C-catalyzed isoflavone biosynthesis. In *cultivar Williams 82*, Xia et al. demonstrated that GmCYP93A1 and GmCYP93A2 both catalyze the conversion of 3,9-dihydroxypterocarpan to glycinol and daidzein. Notably, GmCYP93A1 also directly converts liquiritigenin to daidzein, revealing an alternative branch in the flavonoid metabolic pathway. Importantly, GmCYP93A2 exhibits higher catalytic efficiency than GmCYP93A1 in producing daidzein (Xia et al., 2023). CYP71D8 and CYP82A2 are two cytochrome P450 enzymes that catalyze the final, committed cyclization step in soybean glyceollin biosynthesis, converting 4-dimethylallylglycinol to glyceollin I and 2-dimethylallylglycinol to glyceollin III, respectively (Akashi et al., 2025). Collectively, these findings underscore the pivotal role of cytochrome P450 enzymes in shaping legume-specific isoflavone biosynthetic pathways and reveal novel catalytic mechanisms that advance our understanding of isoflavonoid formation.

### 2.4 CYPs regulate alkaloid biosynthesis

Alkaloids are a prominent class of nitrogen-containing natural products, with structures ranging from simple monocyclic to highly complex polycyclic frameworks. Indole, steroidal, terpenoid, isoquinoline, and dibenzylisoquinoline alkaloids exhibit significant anti-inflammatory and antitumor activities in both human and animal models, underscoring their broad therapeutic potential (Oladeji et al., 2024). The cytochrome P450 family, with its catalytic versatility and organ-specific expression, serves as a dual regulatory system governing both the structural diversification and spatial organization of alkaloid biosynthetic pathways in plants.

#### 2.4.1 Oxidative modification reactions catalyzed by CYPs

The cytochrome P450 family regulates alkaloid accumulation through two principal mechanisms: oxygen atom insertion into the terpenoid backbone and compartmentalized gene expression in specific plant organs. These oxidative modifications introduce functional groups that serve as substrates for downstream enzymes, including reductases, methyltransferases, and glycosyltransferases, collectively enabling the biosynthesis of bioactive alkaloids. Li et al. identified CYP710 genes on chromosome 18 in *Dendrobium chrysotoxum*, *D. catenatum*, and *D. huoshanense*, which are predicted to catalyze sterol C-22 desaturation. More notably, in *D. nobile*, the CYP72 family members DnoNew43 and DnoNew50 form a tandem gene cluster on chromosome 5 and show markedly higher expression in stems than in leaves. This stem-predominant expression suggests that these enzymes catalyze oxidative modifications of the terpenoid scaffold, thereby channeling metabolic intermediates toward dendrobine biosynthesis via stem-specific pathways (Li et al., 2024). A comparable role has been proposed for CYP71 family members in *Jacobaea*, where they are thought to contribute to the structural diversification of alkaloids (Chen et al., 2020). More importantly, CYP80F1 is a cytochrome P450 enzyme that functions as a key mutase/monooxygenase in tropane alkaloid biosynthesis. It stereospecifically catalyzes the oxidative rearrangement of (R)-littorine to form hyoscyamine aldehyde, a direct precursor to hyoscyamine, while also exhibiting hydroxylase activity to produce 3′-hydroxylittorine. Its identification resolves a long-standing mechanistic question in the pathway and enables future metabolic engineering efforts (Li et al., 2006).

#### 2.4.2 CYPs are involved in hydroxylation reactions

Hydroxylation represents the primary reaction through which cytochrome P450 enzymes introduce structural diversity into alkaloids. In *Camptotheca acuminata*, CaCYP72A565 catalyzes two sequential transformations: the hydroxylation of 7-deoxyloganic acid at C-7 to form loganic acid, followed by cleavage of the C7–C8 bond in loganic acid or loganin to yield secologanic acid or secologanin, respectively. Subsequently, the class II CYP enzyme CaC10H (camptothecin 10-hydroxylase), activated by a light-dependent electron transfer system, introduces a hydroxyl group at the C-10 position of camptothecin to form 10-hydroxycamptothecin—a metabolite with significantly enhanced bioactivity (Wei et al., 2025). Thus, site-specific hydroxylation not only modifies the alkaloid scaffold but also enhances its pharmacological efficacy.

In *Eschscholzia californica*, EcCYP82 catalyzes the C-10 hydroxylation of dihydrosanguinarine to form 10-hydroxydihydrosanguinarine. In contrast, EcCYP82P3 catalyzes the same C-10 hydroxylation on both dihydrosanguinarine and dihydrochelerythrine, yielding 10-hydroxydihydrosanguinarine and 10-hydroxydihydrochelerythrine, respectively (Hori et al., 2018). These enzymatic transformations highlight the substrate specificity and positional precision of CYP-mediated hydroxylation, enabling the biosynthesis of structurally distinct alkaloid derivatives from common precursors.

#### 2.4.3 Metabolic networks of multi-enzyme synergy

Cytochrome P450 enzymes exhibit stringent positional and stereochemical selectivity, sequentially acting on a single substrate to build polyhydroxylated scaffolds with specific biological activities. In *Morus alba*, MaCYP82C169 initiates this enzymatic cascade by hydroxylating the C-7 methyl group of (R)-2-methylpiperidine to yield (S)-2-hydroxymethylpiperidine. This intermediate is then hydroxylated at the 4-position by MaCYP71BG22, forming (2R,4R)-2-methylpiperidin-4-ol and thus completing the key polyhydroxylation steps in the biosynthesis of 1-deoxynojirimycin (DNJ) (Fan et al., 2025). It not only enhances our understanding of the origins of diversity and complexity in plant secondary metabolism but also provides a crucial molecular foundation and research targets for deciphering complete biosynthetic pathways of important natural products, exploring plant chemical defense mechanisms, and enabling efficient biomanufacturing through synthetic biology approaches.

#### 2.4.4 CYP enzyme-catalyzed isomerization reaction

Beyond oxygen atom insertion, cytochrome P450 enzymes catalyze more complex reactions, including non-oxidative isomerizations. In *Rauvolfia serpentina*, CYP82S18 not only hydroxylates its substrate but also mediates the non-oxidative rearrangement of vomilenine to perakine, thereby initiating a metabolic branch point leading to a distinct class of alkaloids (Dang et al., 2017). This dual functionality underscores the enzymatic versatility of CYPs and significantly expands the structural and biological diversity of plant-derived alkaloids.

#### 2.4.5 Alkaloid skeleton rearrangement reactions undergo complex chemical modifications

Cytochrome P450 enzymes also mediate intricate skeletal rearrangements that give rise to novel alkaloid scaffolds. In *Hordeum vulgare*, the CYP76 family member CYP76M57 catalyzes a key rearrangement during gramine biosynthesis: it oxidizes the C2 side chain of tryptophan, initiating the sequential loss of the carboxyl group and the α-carbon, coupled with repositioning of the C–N bond. This results in migration of the nitrogen atom from Cα to Cβ, forming a new Cβ–N bond to yield 3-aminomethylindole (AMI), the immediate precursor to gramine (Ishikawa et al., 2024). This CYP-mediated skeletal remodeling underscores the pivotal role of P450 enzymes in plant secondary metabolism and provides crucial molecular insights into the mechanisms of chemical defense and natural product biosynthesis (**Table 1**).

**Table 1:** Representative CYPs involved in plant secondary metabolic biosynthesis and their functions.

Plant species	Secondary metabolites	CYPs	Substrate	Product	Reaction	Function
*Gossypium hirsutum*	Terpenes	CYP71BE79	Gossypol precursor	Gossypol	Oxidation	Participate in disease defense responses (Han et al., 2022)
*Zea mays L.*	Terpenes	CYP71Z18	Dolabradiene	Zealexins	Oxidation	Enhance antimicrobial capability (Mao et al., 2016)
*Glycyrrhiza uralensis*	Terpenes	CYP716A179	triterpenoid β-amyrin	glycyrrhizic acid	Oxidation	A key enzyme for the production of glycyrrhizic acid (Ghosh, 2017)
*Tripterygium wilfordii*	Terpenes	CYP712K1/K2/K3	Friedelin	Tripteroid precursor	Hydroxylation, Oxidation	Promotes the production of anti-cancer substances (Pei et al., 2021)
*Artemisia annua*	Terpenes	CYP71AV1	Amorpha-4,11-diene	Artemisinic acid	Oxidation	Produces a precursor to artemisinin (Chen et al., 2021)
*Camptotheca acuminata*	Terpenes	CYP716	Oleanolic acid	Key intermediates	Hydroxylation	Promoting the Synthesis of Pentacyclic Triterpenoids (Pu et al., 2023)
*Salvia miltiorrhiza*	Terpenes	CYP76AH1	Miltiradiene	Tanshinone precursor	Hydroxylation	Generation of Danshenone Compounds (Mao et al., 2020)
*Medicago truncatula*	Terpenes	CYP93E2	β-amyrin	Sapogenol	Hydroxylation	Key saponin synthase (Confalonieri et al., 2021)
*Panax ginseng*	Terpenes	CYP716C	Dammarenediol-II	PPD, PPT	Hydroxylation	Catalyzing ginsenoside synthesis (Gwak et al., 2019)
*Vitex agnus-castus*	Terpenes	CYP76AH54, CYP71D180	Clerodane-type diterpene precursors	Vitexilactone	Hydroxylation	Generate anti-inflammatory active diterpenes (Heskes et al., 2018)
*Taxus*	Terpenes	CYP725A	Taxadiene	Taxadien-5α-ol	Hydroxylation	Synthesis of paclitaxel precursor compounds (Jennewein et al., 2003; Rontein et al., 2008)
*Solanum tuberosum*	Flavonoids	CYP71	/	Flavonoid compounds	Hydroxylation	Determines tuber color, regulates flavonoid synthesis, and participates in anthocyanin branching (Li et al., 2024)
*Pyrus* spp.	Flavonoids	CYP75	Dihydrokaempferol, Kaempferol	Ihydroquercetin, Quercetin	Hydroxylation	Speed limit steps (Zhang et al., 2023)
*Scutellaria baicalensis*	Flavonoids	CYP82D	Isoflavones	Baicalin, Wogonin	Hydroxylation	Drive the diversity of pharmaceutical ingredients (Qiu et al., 2025)
*Citrus* spp.	Flavonoids	Ciclev10019637m	Multiple flavonoid substrates	3’-Hydroxylated Flavone	Hydroxylation	Key flavonoid synthase (Liu et al., 2023)
*Epimedium sagittatum*	Flavonoids	CYP75A/B	B-ring substrate	Cyanidin- and delphinidin-type anthocyanins	Hydroxylation	Regulating anthocyanin diversity (Huang et al., 2012)
*Camellia sinensis*	Flavonoids	CYP75A/B	4’-Hydroxylated Substrate	3’,4’-Hydroxylated product	Hydroxylation	Participates in the metabolism of flavonoid compounds (Guo et al., 2019)
*Glycine max*	Flavonoids	CYP93C2	Flavone substrate	Isoflavone compounds	Hydroxylation	Aryl Transfer in Isoflavone Biosynthesis (Sawada and Ayabe, 2005)
*Arabidopsis and tomato*	Alkaloids	CYP710	Sterol	C-22 desaturated sterol	Desaturation	Factors affecting the formation of dendrobium alkaloids (Li et al., 2024)
*Camptotheca acuminata*	Alkaloids	CaCYP72A565, CaC10H	7-deoxyloganic acid	Strychnine acid, Secologanic acid	Hydroxylation	Drive the generation of key alkaloids (Wei et al., 2025)
*Eschscholzia californica*	Alkaloids	EcCYP82	Dihydrosanguinarine	10-hydroxydihydrosanguinarine	Hydroxylation	Regulate alkaloid synthesis (Hori et al., 2018)
*Morus alba*	Alkaloids	MaCYP82C169, MaCYP71BG22	(R)-2-methylpiperidine	(S)-2-hydroxymethylpiperidine	Hydroxylation	Constructing a polyhydroxy molecular framework (Fan et al., 2025)
*Rauvolfia serpentina*	Alkaloids	CYP82S18	Vomilenine	Perakine	Isomerization, Hydroxylation	Generate novel structural alkaloids (Dang et al., 2017)
*Hordeum vulgare*	Alkaloids	CYP76M57	Tryptophan	AMI	Skeletal Reconstruction	Drive Skeletal Remodeling (Ishikawa et al., 2024)

### 2.5 CYPs regulate the synthesis of other metabolites

Phenylpropanoids, defined by a C6–C3 skeleton, are widespread secondary metabolites with multiple physiological functions in plants. They contribute to cell wall reinforcement, UV protection, defense against herbivores and pathogens, and floral pigmentation for pollinator attraction. Additionally, phenylpropanoids exhibit diverse bioactive properties beneficial to human health, underscoring their potential for natural product development (Deng and Lu, 2017; Zhu et al., 2024).

Lignin monomers, key end-products of the phenylpropanoid pathway, are synthesized through a rate-limiting 3-hydroxylation step catalyzed by the cytochrome P450 subfamily CYP98. Beyond lignin biosynthesis, CYP98 enzymes also orchestrate carbohydrate and fatty acid metabolism, hormone biosynthesis, and signaling in primary metabolism, thereby serving as central regulatory hubs in the plant metabolic network (Xin et al., 2023). Lignin, a complex polymer, is primarily formed by the polymerization of phenylpropanoid monomers. For example, in *Arabidopsis thaliana*, CYP98A3 specifically catalyzes the 3-hydroxylation of 4-coumaroyl-shikimate to form caffeoyl-shikimate, thereby bridging the upstream phenylalanine metabolism and downstream lignin synthesis pathways at the molecular level. The homologous enzyme CYP98A27 in *Populus trichocarpa* performs the same 3-hydroxylation function, participating in lignin biosynthesis (Alber et al., 2019). CYP98A20 and its close homologs (e.g., CYP98A35) exhibit strict substrate specificity by hydroxylating the p-coumaroyl and tyrosol moieties of osmanthuside B to yield verbascoside. Furthermore, CYP98 enzymes convert p-coumaroyl shikimate or quinate into caffeoyl derivatives, representing key steps that channel carbon flux through the phenylpropanoid network (Yang et al., 2023).

### 2.6 Some specific types of reactions involving CYPs

The Baeyer-Villiger oxidation is a classic method for directly converting aliphatic or cyclic ketones into the corresponding esters or lactones, and the reaction occurs under acidic conditions provided by peracids or hydrogen peroxide. The Baeyer-Villiger oxidation involves the electrophilic attack of a peracid on the carbonyl group of a ketone, forming the key Criegee intermediate; subsequently, this intermediate undergoes a rearrangement in which an alkyl group or hydrogen migrates to the oxygen atom, thereby inserting an oxygen atom between the carbonyl carbon and the adjacent carbon atom, ultimately forming an ester or lactone (Fatima et al., 2024). Takahito Nomura and colleagues analyzed tomato CYP85A1 loss-of-function mutants, and, combined with heterologous expression in yeast and double mutant experiments in Arabidopsis, they for the first time elucidated that the cytochrome P450 enzymes CYP85A3 and CYP85A2 not only catalyze the C-6 hydroxylation reaction in brassinosteroid biosynthesis, but can further convert ketone intermediates into highly active final products with a seven-membered lactone ring, brassinolides, through Baeyer-Villiger oxidation. This reveals a novel enzymatic function of the plant P450 family in catalyzing lactone ring formation and its specific regulatory role in organ development (Nomura et al., 2005).

Rina Saito found that the CYP94 family of monooxygenases (especially SICYP94B18 and SICYP94B19) are mainly responsible for the catabolism of jasmonoyl-isoleucine in tomato leaves. They sequentially convert jasmonoyl-isoleucine into 12-hydroxy-jasmonoyl-isoleucine and 12-carboxy-jasmonoyl-isoleucine through a two-step oxidation reaction, thereby weakening the jasmonate signal and preventing its prolonged activation from harming the plant. In addition, these two enzymes can also catalyze the oxidative metabolism of other jasmonate-amino acid conjugates, indicating their broad role in jasmonate signal regulation. The metabolites they produce are oxidative products of jasmonate plant hormones, specifically hydroxylated and carboxylated derivatives generated via the ω-hydroxylation pathway (Saito et al., 2024).

Mining and studying specialized multifunctional CYPs is of vital importance for a profound understanding and efficient exploitation of plant secondary metabolic networks. These enzymes are not only key links in elucidating the complete biosynthetic pathways of high-value natural products, but also fundamental drivers promoting innovation in natural product science, synthetic biology, and agricultural biotechnology from the very origin.

### 2.7 Conservatism analysis and cross-pathway comparison

CYPs are capable of performing a wide variety of oxidation reactions, based on a highly conserved protein structural framework and catalytic core. As mentioned in the introduction, motifs in the heme-binding domain, such as FxxGxRxCxG and ExxR, are retained in the vast majority of plant CYPs, ensuring the fundamental ability to correctly bind the heme cofactor, activate oxygen, and position substrates. This structural conservation allows different CYP family members, even when involved in different metabolic pathways, to follow similar catalytic cycles. For example, members of the CYP71 family can oxidize diterpene precursors to produce defense metabolites in maize, as well as oxidize indole substrates to participate in camalexin synthesis in Arabidopsis; fundamentally, both processes rely on using a high-valent iron-oxo reactive intermediate to selectively attack inert C-H bonds. Similarly, subfamilies such as CYP76 and CYP716 have been reported to primarily catalyze hydroxylation reactions in terpene, flavonoid, and even alkaloid pathways.

The evolutionary expansion and functional innovation of the cytochrome P450 gene family are key drivers of the diversification of plant secondary metabolites and lineage-specific adaptive strategies. Comparing CYP-mediated metabolic networks in monocots and dicots can reveal both their conserved catalytic logic and the uniquely differentiated defense niches. In terpene biosynthesis, monocots such as *Zea mays* L. rely on the CYP71Z subfamily to catalyze the production of zealexin terpenoids, which exhibit specific antifungal activity in grasses. In contrast, dicots utilize a more diverse array of CYP families, such as CYP71AV, CYP76AH, and CYP716, to construct structurally complex medicinal terpene backbones, such as artemisinin CYP71AV1 in *Artemisia annua* and ginsenoside CYP716A in *Panax*, whose functions extend from direct defense to interactions with mammalian systems. Flavonoid metabolic differences are also notable. Members of the CYP75 family in monocots primarily participate in B-ring hydroxylation of basic flavonoids to respond to abiotic stress. In dicots like *Camellia sinensis*, the CYP75A/B subfamily has further diverged, finely regulating the lineage-specific production of anthocyanins and catechins, directly affecting flower color, flavor, and antioxidant capacity, demonstrating co-evolution with insect pollination and seed dispersal strategies.

As a ubiquitous enzyme family, plant cytochrome P450s (CYPs) participate in nearly all major metabolic pathways (Fang et al., 2024). The catalytic versatility of the CYP98 family and other CYP450s illuminates the organization and regulation of complex metabolic networks. These insights provide a theoretical foundation for the targeted discovery of bioactive compounds and offer practical strategies for enhancing crop resilience through metabolic engineering.

## 3 Molecular mechanisms of CYP gene family regulation in secondary metabolite biosynthesis in plants

CYPs are core functional enzymes in plant secondary metabolism, executing rate-limiting catalytic steps while being tightly regulated by transcription factors, environmental signals, and metabolic feedback. Here we try to clarify the molecular mechanisms controlling CYP activity (rather than framing CYPs as “regulators”) and integrate novel insights into understudied reactions and cross-pathway functional conservation.

### 3.1 Transcriptional and post-transcriptional regulation of *CYP* functional genes

#### 3.1.1 Direct regulation

Transcription factors (TFs) are primary regulators of cytochrome P450 gene expression. *CYP* gene promoters harbor multiple cis-regulatory elements that serve as transcription factor binding sites, enabling the integration of developmental and environmental signals into precise transcriptional responses. WRKY33, a central regulator of plant defense responses, exemplifies this mechanism. WRKY proteins typically recognize the W-box (TTGACC) motif in the promoters of rate-limiting biosynthetic genes. Upon phosphorylation by MPK3/MPK6, activated WRKY33 binds to W-box elements upstream of PAD3 and CYP71A13, two key genes in the camalexin biosynthetic pathway, leading to their transcriptional upregulation (Li et al., 2022). In contrast, WRKY transcription factors can also exert repressive effects. In *Arabidopsis*, WRKY18 and WRKY40 negatively regulate glucosinolate (GLS) biosynthesis. In wrky18 wrky40 double mutants, the transcript levels of CYP81F2, a key gene in GLS biosynthesis, are significantly elevated. This observation indicates that WRKY18 and WRKY40 normally repress CYP81F2expression, likely through direct or indirect binding to its promoter region (Li et al., 2021). A comparable dual-mode regulatory mechanism is evident in the biosynthesis of artemisinin. The AP2/ERF transcription factor AaORA directly activates the expression of CYP71AV1, a downstream rate-limiting enzyme in the pathway. In contrast, the R2R3-MYB repressor AaMYB15 indirectly suppresses CYP71AV1 transcription by inhibiting the activity of AaORA. Notably, silencing AaMYB15 results in increased CYP71AV1 expression and enhanced artemisinin accumulation (Wu et al., 2021). Collectively, these examples demonstrate that transcription factors regulate *CYP* genes in a spatiotemporally controlled and dose-dependent manner, through either direct binding to cis-elements or hierarchical regulatory cascades. This establishes a molecular framework underpinning the metabolic plasticity of plant secondary metabolism.

#### 3.1.2 Co-regulation

Transcription factors (TFs), including MYBs, bHLHs, and GhMYC2, regulate cytochrome P450 gene expression through complex mechanisms involving combinatorial assembly, cis-element binding, and interactions with auxiliary regulatory proteins. This multi-layered regulatory system elucidates the molecular control of flavonoid and gossypol biosynthesis and identifies potential targets for pathway engineering to develop elite germplasm with improved metabolic traits. Chen et al. demonstrated that MYB and bHLH proteins are key regulators of flavonoid biosynthetic genes. The expression of CYP75B1, which catalyzes 3′-hydroxylation of the flavonoid B-ring, peaks at the S4 developmental stage (JNS4) in the JN cultivar. Its expression pattern is positively correlated with that of bHLH2 and MYB5, suggesting coordinated activation by an MYB–bHLH transcriptional complex (Chen et al., 2023). In cotton, GhMYC2 binds to G-box motifs in the promoters of CDNC, CYP706B1, DH1, CYP82D113, and CYP71BE79, enhancing their transcription and promoting gossypol accumulation. However, this activation is antagonized by GhJAZ2, which represses GhMYC2-mediated activation of CYP71BE79. Furthermore, GhMYC2 physically interacts with GoPGF. These findings collectively reveal a complex regulatory network underlying GhMYC2-dependent control of gossypol biosynthesis (Han et al., 2022).

### 3.2 Environmental stress response

Environmental stresses profoundly affect the activity of key enzymes in secondary metabolite biosynthesis (Guo et al., 2025). Under stress conditions, the expression of cytochrome P450 genes is precisely modulated to reprogram secondary metabolic pathways, thereby enhancing plant adaptability and stress tolerance.

#### 3.2.1 Natural stress

In *Ocimum tenuiflorum*, exposure to cold, waterlogging, and drought induces pronounced differential expression of CYP450 genes. Members of the CYP76, CYP75, and CYP716 families show the most robust responses across all stress treatments and are primarily involved in flavonoid and triterpenoid metabolism. Notably, CYP76 family genes display the highest induction under waterlogging stress, underscoring their central role in adapting to this condition. CYP75B1, which catalyzes flavonoid 3′-hydroxylation, may enhance stress resistance by modulating flavonoid biosynthesis to increase antioxidant capacity. Similarly, the triterpenoid-associated CYP716A gene may contribute to stress tolerance through its upregulation and promotion of triterpenoid synthesis. Under drought stress, by specifically regulating the CYP450 gene network, on one hand, the biosynthesis of metabolites with physical and chemical defense functions is directly driven; on the other hand, the homeostasis of key defense hormones, such as JA, BR, and ABA, is precisely modulated. This CYP-mediated diversification of metabolites, together with hormonal signaling programming, collectively forms the plant’s molecular-level adaptive responses, ultimately manifesting as enhanced barrier functions, disease and pest resistance, and optimized resource allocation, thereby improving its defensive resilience and fitness in changing ecosystem (Rastogi et al., 2020).

#### 3.2.2 Salt stress

Population-divergent regulation of CYP450 genes under salt stress has been observed in *Glycyrrhiza glabra*. Exposure to 100 mM NaCl induces CYP88D6—a key gene in glycyrrhizin biosynthesis—in both Fars and Khorasan populations. In contrast, CYP72A154 exhibits population-specific expression: it is upregulated in the Fars population but downregulated with increasing salt concentration in the Khorasan population. These findings suggest that moderate salt stress promotes glycyrrhizin accumulation by activating biosynthetic genes, whereas severe salt stress may suppress it. It also has antioxidant activity, which can help plants alleviate salt-induced oxidative damage. This differential regulation highlights population-specific adaptive networks in response to salinity (Behdad et al., 2020).

#### 3.2.3 Light

Interactions between transcription factors (TFs) and environmental signals add further complexity to gene regulation. Studies indicate that AaMYB15 expression is induced by darkness and jasmonic acid (JA), whereas AaORA activation depends on JA signaling. The resulting AaMYB15–AaORA module integrates light–dark cycles and JA signals to dynamically balance the expression of CYP71AV1, a key gene in artemisinin biosynthesis (Wu et al., 2021).

**Figure 5:**
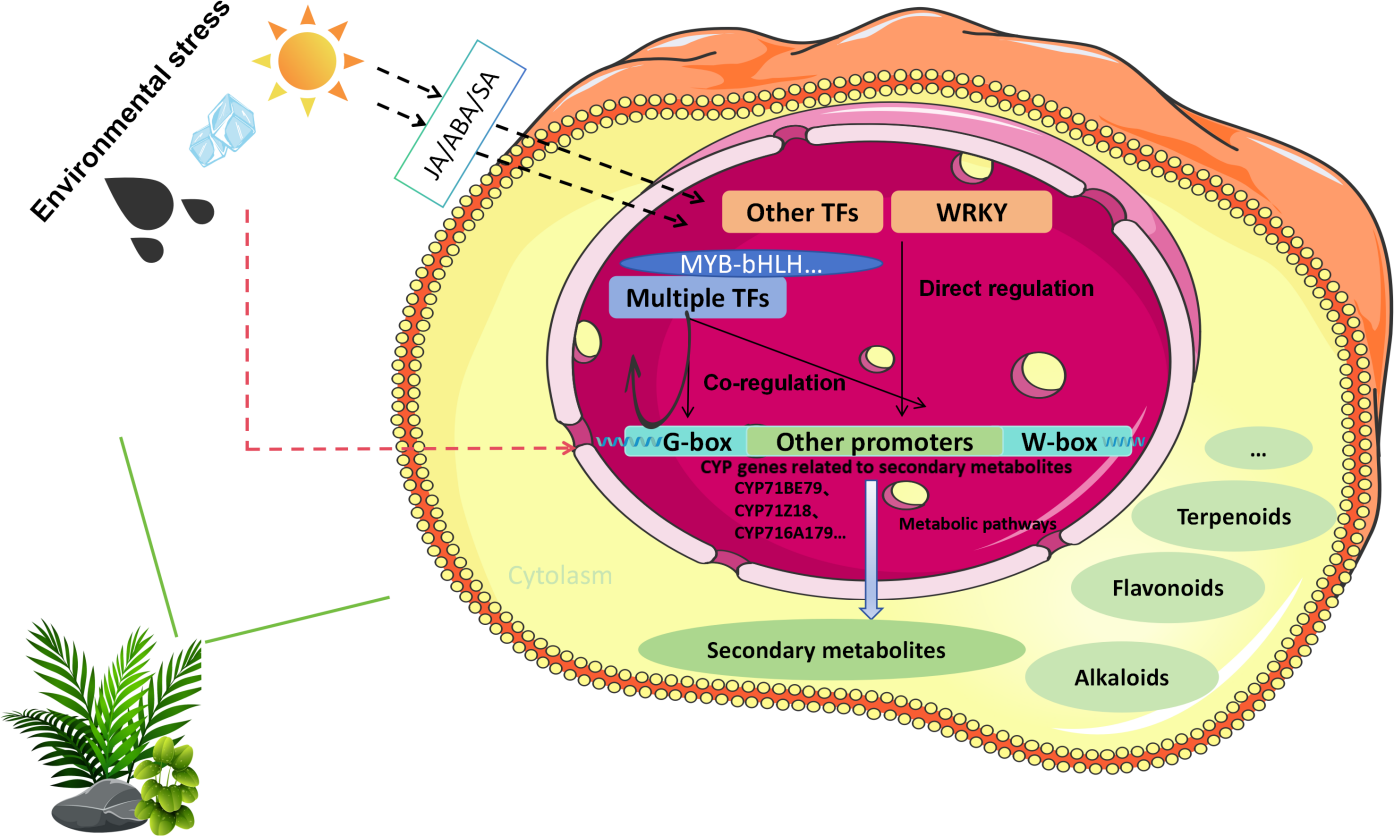
Molecular regulatory mechanisms of the cytochrome P450 gene family in plant secondary metabolite biosynthesis. A, jasmonic acid; ABA, abscisic acid; SA, salicylic acid; WRKY, WRKY transcription factor; MYB-bHLH, MYB-bHLH transcription complex; G-box/W-box, cis-regulatory elements in CYP promoters.

In summary, these studies delineate a multi-tiered regulatory network (**Figure 5**) in which environmental stress directly or indirectly modulates CYP450 expression and enables precise reprogramming of secondary metabolism through transcription factors and their cooperative actions. This sophisticated regulatory architecture constitutes a core adaptive strategy that improves plant stress tolerance. Deeper insight into these mechanisms will advance our understanding of plant stress resistance and open new avenues for developing stress-resilient crops and optimizing the production of bioactive compounds in medicinal plants.

## 4 Prospect

Current research on plant secondary metabolism primarily focuses on elucidating biosynthetic pathways to understand how specific compounds are synthesized and structurally defined. The cytochrome P450 family, one of the most extensive enzyme families in plants, has played a pivotal role in plant evolution and metabolic diversification by catalyzing a broad array of oxygenation and hydroxylation reactions (Srivastava et al., 2015; Urlacher and Girhard, 2019). Ongoing studies are now aimed at deciphering the regulatory networks that govern *CYP* gene expression, where transcription factors, environmental signals, and metabolic pathways integrate into complex regulatory systems. A comprehensive understanding of these interactions is essential for elucidating the mechanisms that control metabolic flux. Equally important is the objective of enhancing the yields of valuable secondary metabolites in agricultural crops through targeted metabolic engineering and breeding strategies.

### 4.1 Deepening the molecular mechanism of CYPs regulation

The cytochrome P450 family plays a central role in the biosynthesis of plant natural products. However, limited understanding of substrate selectivity, structural dynamics, and enzyme–enzyme interactions continues to hinder the development of efficient heterologous production systems. In a study focusing on CYP76AH1—a key enzyme involved in tanshinone biosynthesis—Shi et al. employed directed evolution to modify residues within the substrate-binding pocket, significantly enhancing substrate affinity. Further optimization of the oxygen-binding motif, guided by naturally occurring sequence variations, led to improved catalytic efficiency (Shi et al., 2021). This work presents a clear strategy for the rational engineering of CYP enzymes.

Despite these advances, the majority of plants CYPs remain functionally uncharacterized, and the mechanisms underlying substrate recognition and conformational changes are still poorly understood. Combining high-resolution structural techniques such as cryo-electron microscopy and X-ray crystallography with machine learning models capable of predicting substrate specificity and turnover rates is expected to provide a robust foundation for targeted CYP engineering.

Equally critical is the need to understand how CYP enzymes function within complex and dynamic metabolic networks that mediate plant responses to environmental stress. In high-value biosynthetic pathways such as that of artemisinin, optimizing CYP expression to enhance metabolic flux and compound accumulation remains a major research objective. Future investigations should integrate multi-omics approaches—including transcriptomics and metabolomics—to characterize the tissue-specific expression profiles and co-expression networks of *CYP* genes under various stress conditions. Such integrative analyses will not only clarify the functional roles of CYPs in plant–environment interactions but also establish a theoretical basis for improving metabolite yields through genetic or environmental modulation.

### 4.2 Industrial application of CYPs

Plant secondary metabolites are extensively utilized in medicine, agriculture, and the food industry; however, their naturally low abundance often fails to meet commercial demands. Therefore, developing scalable bioproduction strategies is critically important.

Bioreactors represent the core hardware of modern biotechnology. These systems maintain plant, animal, or microbial cells *in vitro* under precisely controlled conditions to facilitate biochemical reactions and fermentation processes, earning them the designation as the “heart” of biotechnological production. Designing bioreactor systems around the cytochrome P450 gene family presents a promising approach for the sustainable production of plant-derived natural products. CYP mono-oxygenases catalyze the hydroxylation of C–H bonds, thereby enabling subsequent modifications—such as acylations, methylations, and glycosylations—that enhance structural diversity and bioactivity. CYP monooxygenases have been successfully applied in the industrial biosynthesis of several high-value natural products. For example, by expressing CYP76AH3 and CYP76AK1 from Salvia miltiorrhiza in engineered yeast and combining this with fermentation optimization, efficient synthesis of tanshinone compounds can be achieved, with the target product accounting for more than 92% of the total tanshinones (Guo et al., 2016). Beyond native enzymes, rationally engineered P450 BM3 variants have demonstrated exceptional catalytic selectivity. The triple mutant A82F/F87V/L188Q hydroxylated sesquiterpenic acid at the C-7 position with 94% regioselectivity, while a quadruple mutant (A82F/F87V/T268A/L437Q) achieved 90% regioselectivity and complete stereoselectivity at the C-9 position, providing powerful tools for chiral drug synthesis (Urlacher and Girhard, 2019).

NADPH-cytochrome P450 reductase (CPR) is a structurally conserved diflavin protein, with its N-terminal and C-terminal containing domains derived from bacterial flavodoxin and ferredoxin-NADP^+^reductase, respectively, and is localized to the endoplasmic reticulum through a membrane-anchoring region. Its core function is to sequentially transfer electrons from NADPH via FAD and FMN to the heme center of cytochrome P450, driving catalytic oxidation reactions such as hydroxylation, thus playing a key role in the biosynthesis of plant secondary metabolites, defense responses, and environmental adaptation. Its highly conserved interaction interface allows CPRs from different species to partially complement each other, making it an ideal module for constructing novel biocatalytic systems in synthetic biology (Jensen and Møller, 2010; Wei et al., 2025). Regarding the biosynthesis of the core medicinal saponin aglycone Quillaic acid, this study systematically screened 110 CYP/CPR pairings through a combinatorial optimization strategy and successfully constructed an efficient oxidation cascade network composed of CYP716A12, CYP714E19, CYP72A567 (2 copies), and CYP716A262. This network enabled six-step selective oxidation of the substrate β-amyrin at the C-16, C-23, and C-28 positions. By engineering the endoplasmic reticulum (overexpressing ice2) and using the membrane sterol-binding protein MSBP1 to bring CYP enzymes closer together, QA production in 1L fed-batch fermentation was dramatically increased from the previously reported 314 mg/L to 2.23 g/L (approximately a 7.1-fold increase), with a 32-fold increase in specific productivity. Additionally, the purity of QA among oxidized triterpene products rose from 54% to 86%. This achievement highlights the core value of rationally designing and combinatorially optimizing the CYP gene family in achieving efficient, high-purity, and scalable biomanufacturing of complex natural products (Yang et al., 2023). In *Artemisia annua*, six key enzyme genes in the artemisinin biosynthesis pathway—IDI, FPS, ADS, CYP71AV1, AACPR, and DBR2—were co-introduced into the plants, and two core optimization techniques were employed: First, the cytochrome P450 monooxygenase (CYP71AV1) was co-expressed with its specific electron donor, cytochrome P450 reductase (AACPR/CPR), to provide an efficient redox catalytic cycle, addressing the common issue of insufficient reducing power when P450 enzymes are heterologously expressed; second, key enzymes were precisely modified with signal peptides to achieve subcellular compartmentalization. The ADS enzyme was targeted to mitochondria rich in the precursor FPP using the mitochondrial signal peptide cox4, while CYP71AV1, AACPR, and DBR2 were directed to the chloroplasts with chloroplast transit peptides. This non-glycosylated environment not only stabilizes enzyme activity but also prevents futile cycling of metabolic intermediates and potential toxicity in the cytoplasm. This combined strategy synergistically optimized precursor supply, catalytic efficiency, and the spatial environment for product accumulation, ultimately resulting in transgenic A. annua plants with artemisinin content reaching 2.69% of dry weight in the T2 generation—an increase of up to 232% compared to the wild type (0.81%)—and confirmed that this high-yield trait can be stably inherited according to Mendelian genetics in the next generation (Qamar et al., 2024).

Plant CYPs are key drivers of secondary metabolite diversification, catalyzing hydroxylation and epoxidation reactions that enhance the structural complexity and bioactivity of terpenoids, alkaloids, and flavonoids. Suspension culture complements bioreactor-based strategies by propagating callus or somatic embryos in liquid medium on orbital shakers until stable, homogeneous cell lines are established. Supplementing the culture with CYP-inducing elicitors further promotes metabolite accumulation (Bhatia and Bera, 2015). Coupling these cultures with an enhanced NADPH supply—supported by the pentose phosphate pathway and ATP-dependent electron transfer—facilitates the scaling from laboratory to industrial production (**Figure 1**). Integrating advanced bioreactor systems with CYP-centered metabolic engineering effectively circumvents the natural scarcity of plant secondary metabolites. This combined strategy significantly boosts product yield and scalability, improves economic viability across healthcare, agriculture, and green industries, and pushes the field of plant biotechnology to new frontiers.

### 4.3 Application of CYP gene family in breeding and improving the quality of medicinal plants

The cytochrome P450 enzyme system is a core catalytic component in synthetic biology for constructing biosynthetic pathways of medicinal compounds, playing an irreplaceable role in the synthesis of key products such as artemisinin, taxol precursors, and ginsenosides. However, its engineered application still faces numerous bottlenecks: strict substrate specificity limits the expansion of catalytic scope; electron transfer efficiency dependent on CPR is low; protein folding and localization issues often occur in heterologous expression; and during large-scale fermentation, enzyme activity is unstable and by-product accumulation is common.

Salicylic acid (SA) is a key phytohormone regulating pathogen defense responses in plants. Substantial evidence shows that cytochrome P450 mono-oxygenase inhibitors suppress SA biosynthesis across a wide range of plant species (Wang et al., 2025). Molecular breeding strategies now employ targeted pathway engineering—through transcriptional or enzymatic modulation of *CYP* genes—to enhance the accumulation of high-value bioactive compounds in medicinal plants and fungi, while concurrently improving agronomic traits such as stress tolerance. Environmental stress acts as a potent inducer of secondary metabolism, enhancing plant resilience and metabolite biosynthesis both, thereby creating novel opportunities for trait improvement via metabolic engineering. CRISPR/Cas9 technology has significantly advanced these efforts. Shang et al. developed the first functional CRISPR-Cas9 system in the medicinal fungus *Ganoderma lucidum*. By delivering a Cas9–sgRNA ribonucleoprotein complex via PEG-mediated transformation of spore-derived protoplasts, they successfully disrupted cyp512a3, a key gene in triterpenoid ganoderic acid biosynthesis. The edited strains exhibited substantially reduced ganoderic acid levels, confirming the central regulatory role of cyp512a3. Furthermore, this study identified previously unknown CYPs in the type-II ganoderic acid (GA) biosynthetic pathway originally proposed by Yuan et al., and elucidated their catalytic sequences. This work provides direct evidence that CYP-targeted genome editing can effectively generate high-yield Ganoderma strains (Wang et al., 2020; Khanal et al., 2025).

In addition, artificial intelligence technology provides a new approach for the rational design of CYPs. Machine learning-based sequence-to-function predictions can guide the directed evolution of key residues; deep learning-assisted protein structure modeling can accurately elucidate substrate binding patterns; and integrated metabolic networks and computational models can virtually optimize CYP catalytic performance and metabolic flux distribution in synthetic pathways (Huang et al., 2019; Yadav et al., 2024).

A mechanistic understanding of individual CYP enzymes within secondary metabolic networks is therefore essential for redirecting metabolic flux and achieving sustained production of target compounds. This precision-regulation strategy not only ensures stable yields of medicinal resources but also supports the sustainable and scalable long-term development of plant biotechnology.

### 4.4 Interdisciplinary collaboration and integration with new technologies

Future research on the cytochrome P450 family should prioritize interdisciplinary collaboration, integrating bioinformatics, chemical engineering, molecular biology, and synthetic biology. The integration of genomic, proteomic, metabolomic, and environmental datasets will enable a more comprehensive understanding of the functional diversity and complexity of CYP regulatory networks. When combined with artificial intelligence and advanced big-data analytics, these multidisciplinary approaches will allow for the systematic characterization of CYP functions in plant metabolism. This will provide a robust theoretical foundation and precise tools for the engineered production of valuable secondary metabolites.

Of course, it also raises potential ethical and ecological issues, such as unintended effects on non-target organisms and the possibility of gene flow to wild relatives. We can wait to use methods such as employing tissue-specific promoters to restrict the accumulation of metabolites in the edible parts to address the above issues.

In summary, a systematic analysis of *CYP* genes and their regulatory networks in medicinal plants and fungi is essential for discovering novel therapeutic agents and natural products, as well as for elucidating the roles of secondary metabolites in disease treatment. As our understanding of these regulatory mechanisms deepens, continued scientific and technological advances are poised to accelerate innovations in medicine, ultimately contributing to improved human health.
